# Age- and ethnic-driven molecular and clinical disparity of East Asian breast cancers

**DOI:** 10.1186/s12916-024-03638-y

**Published:** 2024-09-27

**Authors:** Ji Yoon Lee, Ji Won Lee, Min Sung Chung, Jong Gwon Choi, Sung Hoon Sim, Hyo Jeong Kim, Jeong Eun Kim, Kyoung Eun Lee, Yeon Hee Park, Myoung Joo Kang, Mi Sun Ahn, Yee Soo Chae, Ji Hyun Park, Jee Hyun Kim, Gun Min Kim, Jae Ho Byun, Keon Uk Park, Ju Won Kim, Seung Pil Jung, Jung Hyun Lee, Jung Seok An, Byunghyun Jang, Dayoung Yoon, Jiwon Kim, Jisoo Hong, Harim Koo, Kyu Ran Cho, Cheol Yong Kim, Jason K. Sa, Kyong Hwa Park

**Affiliations:** 1 Department of Biomedical Informatics, Korea University College of Medicine, Seoul, Republic of Korea; 2 Department of Biomedical Sciences, Korea University College of Medicine, Seoul, Republic of Korea; 3 Department of Internal Medicine, Division of Medical Oncology, Korea University Anam Hospital, Seoul, Korea; 4 Department of Surgery, College of Medicine, Hanyang University, Seoul, 04763 Korea; 5 Department of Oncology-Hematology, Konyang University Hospital, Daejeon, Korea; 6 Center for Breast Cancer, National Cancer Center, Goyang, Republic of Korea; 7 Department of Internal Medicine, Division of Hematology-Oncology, School of Medicine, Medical Research Institute, Pusan National University Hospital, Busan, Korea; 8 Department of Oncology, Asan Medical Center, University of Ulsan College of Medicine, Seoul, Republic of Korea; 9 Department of Hematology and Oncology, Ewha Womans University Hospital, Seoul, 07985 Republic of Korea; 10 Samsung Medical Center, Sungkyunkwan University School of Medicine, Seoul, Republic of Korea; 11 Department of Internal Medicine, Inje University Haeundae Paik Hospital, Busan, Korea; 12 Department of Hematology-Oncology, Ajou University School of Medicine, Suwon, Korea; 13 Department of Hematology and Oncology, School of Medicine, Kyungpook National University, Kyungpook National University Chilgok Hospital, Daegu, Republic of Korea; 14 Department of Hematology-Oncology, Division of Internal Medicine, KonKuk University Medical Center, Seoul, Republic of Korea; 15 Department of Internal Medicine, Division of Hematology and Medical Oncology, Seoul National University Bundang Hospital, 166 Gumi-Ro, Bundang-Gu, Seongnam, 463-707 Korea; 16 Department of Internal Medicine, Division of Medical Oncology, Yonsei University College of Medicine, 50-1 Yonsei-Ro, Seodaemun-Gu, Seoul, 120-752 Korea; 17 Department of Internal Medicine, Division of Oncology, Incheon St. Mary’s Hospital, College of Medicine, The Catholic University of Korea, Seoul, Republic of Korea; 18 Department of Internal Medicine, Division of Hematology-Oncology, Keimyung University Dongsan Hospital, Keimyung University College of Medicine, Daegu, Republic of Korea; 19 Department of Surgery, Department of Breast Surgery, Division of Breast and Endocrine, Korea University Anam Hospital, Seoul, Korea; 20 Department of Pathology, Korea University Anam Hospital, Seoul, Korea; 21 KU-KIST Graduate School of Converging Science and Technology, Korea University, Seoul, Republic of Korea; 22 Department of Cancer Biomedical Science, Graduate School of Cancer Science and Policy, National Cancer Center, Goyang, South Korea; 23 Department of Radiology, Korea University Anam Hospital, Seoul, Korea

**Keywords:** Breast cancer, Ethnic diversity, Genomic alterations, Molecular subtypes, Precision medicine

## Abstract

**Background:**

Breast cancer (BC) is a complex disease with profound genomic aberrations. However, the underlying molecular disparity influenced by age and ethnicity remains elusive.

**Methods:**

In this study, we aimed to investigate the molecular properties of 843 primary and metastatic BC patients enrolled in the K-MASTER program. By categorizing patients into two distinct age subgroups, we explored their unique molecular properties. Additionally, we leveraged large-scale genomic data from the TCGA and MSK-IMPACT studies to examine the ethnic-driven molecular and clinical disparities.

**Results:**

We observed a high prevalence of *PI3KCA* mutations in K-MASTER HER2 + tumors, particularly in older patients. Moreover, we identified increased mutation rates in DNA damage response molecules, including *ARID1A*, *MSH6*, and *MLH1*. The K-MASTER patients were mainly comprised of triple-negative breast cancer (TNBC) and HER2-positive tumors, while the TCGA and MSK-IMPACT cohorts exhibited a predominance of hormone receptor-positive (HR +) subtype tumors. Importantly, *GATA3* mutations were less frequently observed in East Asian patients, which correlated with poor clinical outcomes. In addition to characterizing the molecular disparities, we developed a gradient-boosting multivariable model to identify a new molecular signature that could predict the therapeutic response to platinum-based chemotherapy.

**Conclusions:**

Our findings collectively provide unprecedented insights into the significance of age and ethnicity on the molecular and clinical characteristics of BC patients.

**Supplementary Information:**

The online version contains supplementary material available at 10.1186/s12916-024-03638-y.

## Background

Breast cancer (BC) is one of the most prevalent malignancies, accounting for one-quarter of all cancer diagnoses in women, resulting in an estimated 680,000 deaths worldwide each year [1]. BC can be classified into distinct subtypes based on unique molecular and histopathological characteristics, namely estrogen receptor-positive (ER +), human epithelial receptor-positive (HER +), and triple-negative breast cancer (TNBC). Personalized treatment approaches tailored to each molecular subgroup have been largely established, highlighting the importance of targeted therapies [2, 3]. The rapid advancements of clinical next-generation sequencing (NGS) technology have revolutionized the field of oncology, enabling the examination of molecular profiles and identification of therapeutic targets across a broad range of different tumor types [4–6]. Notably, large-scale genomic studies, such as The Cancer Genome Atlas (TCGA) and Memorial Sloan Kettering (MSK)-IMPACT, have contributed significantly to our understanding of the impact of genetic alterations on treatment response and prognosis in BC patients [7–13].

While menopause is speculated to be associated with an increased risk of breast cancer development due to hormonal changes, the prevalence of pre-menopausal breast cancer is significantly higher in the East Asian populations compared to the Western cohorts [14, 15]. Furthermore, approximately one-third of all East Asian breast cancer patients are diagnosed between the ages of 40 and 49 which is approximately 10 years earlier than the commonly diagnosed age in European and Western countries [16, 17]. Therefore, there has been growing interest in understanding the incidence and characteristics of pre-menopausal young breast cancer (YBC) patients due to their distinct molecular properties and clinical implications [18]. YBC constitutes a small but significant subset of BC cases. These patients exhibit aggressive biological traits, including frequent metastasis and relapse, as well as a higher likelihood of being diagnosed with hormone receptor-negative tumors, leading to poor clinical outcomes [19, 20]. Notably, the incidence of YBC has been rising, particularly in the East Asian populations [21, 22]. Therefore, significant efforts assessing the molecular disparity between YBC and older breast cancer (OBC) patients have been established. A multi-omics study of primary tumors in YBC patients has suggested that younger Asian BCs were characterized by an immune-active microenvironment [23]. Furthermore, copy number loss in *APOA1/C3/A4/A5* has been suggested as a possible mechanism for abundant immune microenvironment in Asian YBC patients [24]. Additional studies have suggested a difference between YBC and OBC, with *GATA3*, *TP53*, *ARID1A*, and *CTNNB1* mutations enriched in the younger group, while older patients often carried mutations in *CDH1*, *PIK3CA*, and *MAP3K1*. In the ER + subgroup, activation of integrin and laminin signaling pathways and EGFR signaling were considerably enriched in premenopausal patients [25–28]. While these studies are informative, they have yet to thoroughly address the molecular variations across different racial groups, particularly in primary and metastasis settings. Furthermore, the integration of molecular findings with clinical outcomes, a crucial aspect of large-scale genomic studies for translational research, has been lacking. Therefore, the clinical implications of observed molecular variations remain uncertain.

In this study, we aimed to characterize the complex genome of 847 East Asian BC patients enrolled in the K-MASTER program to uncover essential genomic aberrations associated with the unique molecular properties of YBC and OBC patients. Additionally, we investigated ethnic-driven genomic diversity in primary and metastatic BC by leveraging previously established large-scale genomic studies such as TCGA and MSK-IMPACT. Lastly, we integrated clinical attributes, including patient survival and response to platinum-based chemotherapy, to elucidate the profound effects of genetic alterations on clinical outcomes, in hopes of enhancing our understanding of this lethal disease.

## Methods

### K-MASTER tumor specimen collection

We acquired tumor tissue specimens from breast cancer patients participating in the K-MASTER project, which aims to gather and analyze the molecular profiles of 10,000 Korean patients with advanced solid tumors [6]. In total, we collected and analyzed 843 breast cancer tissue specimens, consisting of 656 primary tumors and 191 metastases from 843 patients. To evaluate the treatment response to platinum-based chemotherapy, we analyzed patients who had received either cisplatin or carboplatin in KM and MSK studies. We used RECIST version 1.1 criteria to evaluate the therapeutic efficacy of the treatment. Patients have been categorized as responders if they achieved complete response (CR) or partial response (PR) and as non-responders if they showed stable disease (SD) or progressive disease (PD). Progression-free survival (PFS) was calculated by measuring the time from the beginning of treatment to either the occurrence of disease progression or the date of death. For patients who had not experienced disease progression at the time of the data freeze, their clinical data were censored based on their last follow-up date. Similarly, for patients who were still alive, their data were censored at the date of their last follow-up.

### K-MASTER sequencing panels

In the K-MASTER project, we employed three previously established and validated tissue-based next-generation sequencing (NGS) panels, including FIRST, CancerSCAN, and K-MASTER, to identify significant genomic abnormalities such as mutations, copy number variations (CNVs), and small insertions and deletions in genes associated with cancer. Only the genes that were covered by all three panels were subjected to downstream analysis. Genomic DNA was extracted from formalin-fixed paraffin-embedded (FFPE) samples. Genomic DNA samples meeting the quality control criteria were centrally isolated and then sent to the K-MASTER genomic analysis laboratories for further processing.

### Variant calling

The FASTQ files containing the sequenced reads were aligned using the Burrows-Wheeler Aligner, utilizing the human genome assembly (hg19) as the reference [29]. The resulting alignment files in BAM format underwent several preprocessing steps. These steps included sorting the files by SAMtools, removing duplicated reads by Picard, and recalibrating the base quality scores by Genome Analysis Toolkit (GATK). To ensure accurate mutation identification, we employed MuTect2, which allowed us to make high-confidence predictions on mutation calls. Germline variants were excluded using previous large-scale genomic studies, including the 1000 Genomes Project, Exome Aggregation Consortium (ExAC), and Genome Aggregation Database (gnomAD). Only the variants that were not previously reported or with a population allele fraction below 0.005% across all East Asian subpopulations were retained. A stringent downstream filter was applied to identify high-quality somatic variants, requiring a minimum coverage of 20x, variant allele fraction (VAF) of ≥ 2%, and being labeled as “PASS” in the “FILTER” field. Subsequently, mutations in non-coding regions, such as 3′UTR, 5′UTR, introns, and intergenic regions, were eliminated. Using CNVkit, we acquired the copy number alterations of target genes [30]. CNVkit presents copy number alterations as a log2 ratio change. Copy number variations (CNVs) are reported when the log2 copy number gain > 1 or log2 copy number gain <  − 1.

### Genomic diversity comparison of the K-MASTER, TCGA, and MSK cohorts

To investigate and compare the frequency of major genomic abnormalities based on ethnicity, we obtained somatic mutation data from TCGA and MSK-IMPACT datasets, along with clinical data from the Genomic Data Commons. This analysis only included high-quality somatic mutations that met the criteria and were not classified as germline mutations during the variant calling process. For TCGA, we specifically selected patients annotated as “WHITE” in the “race” column, resulting in a final list of 705 patients. In the case of the MSK dataset, we focused on metastatic samples, resulting in a final list of 873 patients. Further selection was performed by only selecting the genes that were captured by the K-MASTER sequencing panels. All groups were subdivided into YBC and OBC based on age 40 years. The Fisher test was performed to assess the significance of differences in the frequency of mutations and copy number variations at the individual gene level between the K-MASTER and the TCGA and MSK cohorts.

### Mutational signatures

To conduct mutational signature analysis, we employed the deconstructSigs package (version 1.8.0) in R [31]. This analysis involved a set of mutations categorized into six substitution classes, namely C > T, C > A, C > G, T > C, T > A, and T > G. The base contexts immediately preceding and following the mutated nucleotide within the exome regions were also taken into account. In addition, we utilized a collection of 30 reference mutational signatures from the “signature.cosmic” database. These signatures were associated with specific biological processes and were represented by terms such as aging (signature 1), APOBEC (signature 2, 13), DNA-DSBR (double-strand break repair; signature 3), DNA-MMR (mismatch repair; signature 6, 15, 20, 26), UV (signature 7), and others (signature 4, 5, 9, 11, 12, 17, 22, 23, 24, 28). To ensure reliable results, we filtered out mutation signatures that were present in less than 5% of the samples within each tumor type. This filtering step allowed us to focus on the more prevalent mutational signatures within each specific tumor type for further analysis and interpretation.

### Mutual exclusivity analysis

Mutually exclusive driver networks in the K-MASTER (KM) dataset were analyzed using the Mutual Exclusivity Modules in Cancer (MEMo) tool (version 1.0) [32]. The MEMo analysis was conducted following the previously established methods [13]. For the KM samples, alterations affecting at least 1% of the samples were selected. Significant mutually exclusive modules were identified based on an FDR-corrected *p* value threshold of < 0.1.

### Survival analysis

Overall survival (OS) and progression-free survival (PFS) analyses for the KM, TCGA, and MSK datasets were conducted using the “survival” package (version 3.3–1) in R. Kaplan–Meier survival curves were generated to compare prognostic outcomes across different cohorts. To assess differences in survival between categorical variables, the log-rank test was employed. Additionally, standard multivariate Cox proportional hazards modeling was utilized to estimate hazard ratios across molecular subtypes.

### Determination of deleterious mutation status

For our analysis, we classified all loss-of-function alterations as deleterious. This category included mutations such as nonsense mutations, frameshift mutations, and splice site alterations. To assess the functional impact of amino acid substitutions in proteins, we utilized two prediction methods: Polyphen-2 [33] and SIFT [34]. Polyphen-2 employed a classification approach to predict the potential functional impact of missense mutations. Any missense mutations classified as “possibly damaging” or “probably damaging” in Polyphen-2 or “deleterious” in SIFT were considered deleterious. By incorporating the predictions from both Polyphen-2 and SIFT, we identified and included all missense mutations that were classified as deleterious in our analysis, ensuring a comprehensive assessment of potentially harmful alterations in protein function.

### Estimating the therapeutic response to platinum-based chemotherapy

In this study, we utilized XGBoost package (version 1.6.0.1) in R, an algorithm based on gradient tree boosting, to assess the significance of mutations in predicting the response to platinum-based therapy. This analysis was conducted as part of the first module of our proposed approach. The study population was divided into training and testing sets in a 7:3 ratio in a random manner. To determine the importance of mutations, we employed the concept of “gain,” which represents the average feature importance provided by the gradient tree boosting method. This measure allowed us to evaluate the contribution of each mutation to the prediction of chemotherapy response. By utilizing XGBoost and considering the gain values, we generated an initial list of candidate risk-predictive mutations, thereby providing valuable insights into the potential influence of genetic variations on the response to platinum-based therapy.

### Statistical analyses

Differences in mutation frequency and replication number change frequency between cohorts were tested using the Fisher test. All hypothesis tests were conducted as two-sided tests, and statistical significance was determined as a *p* value less than 0.05. The statistical analyses were carried out using R software version 4.0.5.

## Results

### Comprehensive molecular characterization of YBC and OBC reveals significant molecular disparity

**Fig. 1:**
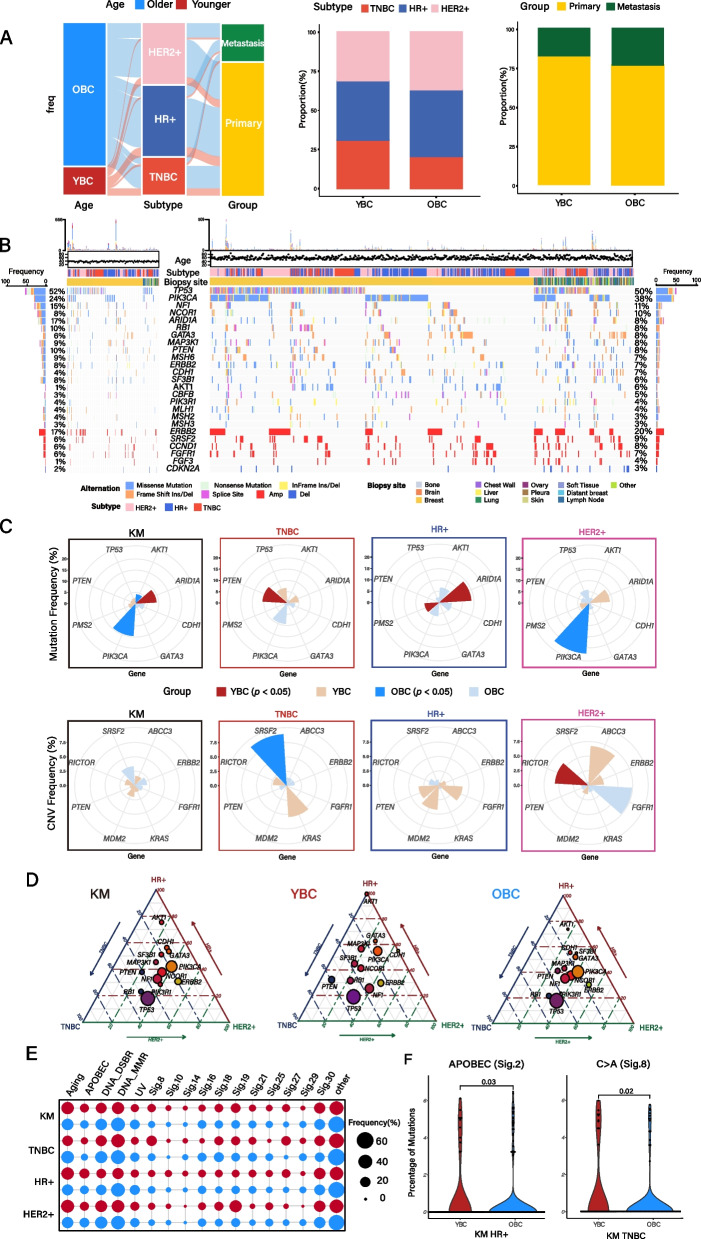
Genomic landscape of KM BRCA. **A** Overall characteristic of KM BRCA sample and clinical data. Clinical features of KM BRCA sample. **B** Genomic landscape of somatic mutations and copy number alterations of BRCA by age group. **C** Significantly mutated genes by age group in KM BRCA. **D** Ternary diagram depicting mutation proportion of all, YBC, and OBC group by molecular subtypes. The size of each node represents the number of tumors with the respective mutations, and the color spectrum indicates its relative frequency. **E** Mutational signature of all and molecular subtype. The size of each dot represents the proportion of samples of each tumor types. The red dot represents the YBC group, and the blue dot represents the OBC group. **F** Violin plots demonstrating signature differences of YBC and OBC group

To uncover the molecular disparity between YBC and OBC, we analyzed target-exome sequencing data from 602 breast cancer patients in our previous publication of the K-MASTER study [6]. Additionally, we performed sequencing on an additional 241 cases, resulting in a total of 843 BC patients. We utilized previously established and validated sequencing panels with sufficient coverage and depth to detect genetic alterations at subclonal levels, including single nucleotide variations, small insertions and deletions, and copy number alterations (CNAs). Based on previous East Asian BC studies that have coherently employed “40-year-old” as an appropriate threshold to classify young breast cancer patients [23, 35], we categorized our cohort into two age groups, YBC (women under the age of 40, *n* = 142) and OBC (women over the age of 40, *n* = 701). We first examined the prevalence of major molecular subtype compositions, including hormone receptor-positive (HR +), HER2 + , and TNBC, between YBC and OBC. Notably, TNBC tumors were more prominent in YBC patients, while HER2 + tumors were more common in OBC patients [36] (Fig. 1A). To examine the essential genomic characteristics according to the molecular subtypes, we conducted a comparative analysis with previous Asian breast cancer cohorts [23, 37]. We discovered that TNBC tumors demonstrated similar mutation frequencies in major driver genes, including *TP53* and *PIK3CA* (Additional file 1: Fig. S1). In the HR + subtype tumors, both K-MASTER and SMC cohorts exhibited high mutational frequencies in *TP53*, *PIK3CA*, and *GATA3*. Lastly, HER2 + tumors showed enriched *TP53* and *PIK3CA* mutations. When assessing the clinical progression stage, the K-MASTER cohort was characterized by a slightly higher proportion of stage IV tumors in OBC patients, particularly in the TNBC subtype (Additional file 1: Fig. S3A).

Among the 843 BC patients, we detected 14,429 nonsynonymous mutations, including missense, nonsense, in-frame, frameshift, and splice-site, and 802 CNAs. The most prevalent somatic mutation was in *TP53*, observed in both YBC and OBC patients. The next frequently altered gene was *PIK3CA*, more commonly found in OBC patients compared to YBC (38% vs. 24%) (Fig. 1B–C). Conversely, YBC patients exhibited a higher degree of *AIRD1A* mutations. Notably, these mutation enrichments were associated with specific molecular subtypes, where *ARID1A* and *PMS2* mutations were predominantly found in HR + tumors, while *PIK3CA* mutations were more prevalent in the HER2 + subtype (Fig. 1C). Other prominent genomic aberrations included *PTEN* mutations and *SRSF2* amplifications in TNBC tumors of YBC and OBC, respectively, as well as amplification of *RICTOR* in HER2 + type YBC tumors (Fig. 1C). We further examined the genomic disparity among distinct molecular subtypes between YBC and OBC. Consistent with previous reports, TNBC tumors were characterized by enrichments of *TP53* and *RB1* mutations in both YBC and OBC patients (Fig. 1D). In the HR + subgroup, mutations in *AKT1*, *CDH1*, *GATA3*, and *MLH1* were frequently identified, while *ERBB2* mutations were more common in the HER2 + tumors. *PTEN* mutation was notably more enriched in TNBC tumors of YBC. As TNBC tumors are often diagnosed at an earlier age compared to other subtypes, we further subcategorized OBC patients into two subgroups, an intermediate breast cancer (IBC) for those between 40 and 60 and an elderly breast cancer (EBC) group for those over 60 (Additional file 1: Fig. S2A–B). As a result, we discovered that EBC patients demonstrated significant enrichments of *RSF1*, *ROS1*, and *TP53BP1* mutations (Additional file 1: Fig. S2C). To identify clinically actionable genetic alterations, we curated the OncoKB knowledge database [38] and identified *AKT1* E17K mutation was highly enriched in OBC HR + tumors. Furthermore, *PIK3CA* H1047R mutations, which confer increased sensitivity to alpelisib and fulvestrant, were predominantly seen in TNBC or HER2 + tumors, suggesting an alternative therapeutic opportunity for older BC patients (Additional file 1: Fig. S3B).

To explore the dynamic interactions among major driver mutations, we employed the Mutual Exclusivity Module in Cancer (MEMo) algorithm [32]. We discovered 34 modules that were significantly enriched in the KM cohort (*q* value < 0.1; Additional file 2: Table S1). The most frequently occurring mutually exclusive modules were related to the PI3K-AKT-mTOR pathway, including the mutations in *ERBB2*, *PIK3CA*, *PIK3R1*, *PTEN*, and *AKT1*. These molecules appeared in an exclusive reciprocal manner not only in the entire breast cancer cohort but also in the YBC and OBC subgroups as well (Additional file 2: Fig. S1A). Moreover, several epigenetic-related genes such as *ARID1A*, *EP300*, and *CREBBP* were also identified, highlighting the functional role of epigenetic modulation during tumor progression. When we delineated the module enrichments based on the molecular subtype, both HR + and HER2 + groups exhibited a prevalence of *EP300*, *AKT1*, and *PIK3CA* mutations, while TNBC and non-TNBC tumors demonstrated a significant difference in mutational patterns within the PI3K-AKT-mTOR pathway (Additional file 2: Fig. S1B). Next, we analyzed the repertoire of mutational signature activities between YBC and OBC patients. Both age groups demonstrated enrichments of mutational signatures associated with age and DNA mismatch repair deficiency [9] (Fig. 1E). Notably, the APOBEC and C > A transition signature activities were predominantly observed in YBC patients, particularly in the HR + and TNBC tumors, respectively (Fig. 1F). Collectively, our results provide profound insights into the molecular disparity between YBC and OBC patients, revealing distinct mutational patterns, subtype composition, and potential therapeutic opportunities.

### Ethnic-driven molecular disparity in primary YBC and OBC patients

Previous studies have underscored the substantial molecular disparities among patients from different ethnic backgrounds [39–41]. In order to systematically compare the unique genomic profiles of primary YBC and OBC based on distinct racial populations, we utilized a comprehensive dataset of large-scale genomic data from TCGA patients diagnosed with primary BC. Consistent with previous findings, K-MASTER primary BC patients were diagnosed at a significantly younger age, with an average of 51.8 years, in contrast to the average age of 58.5 years observed in the TCGA cohort (Additional file 1: Fig. S4A). Notably, the K-MASTER cohort was composed of a higher proportion of the TNBC and HER2 + tumors, while TCGA was predominantly composed of the HR + subtype (Additional file 1: Fig. S4B). This distinction in molecular subtypes was consistently observed in both YBC and OBC patients, although the difference was minimal for the TNBC-type tumors in YBC (Additional file 1: Fig. S4C). A comprehensive analysis of the OncoKB database revealed that TCGA patients exhibited a significant number of clinically actionable mutations, including *AKT1* and *PIK3CA* (Additional file 1: Fig. S4D). However, when evaluating at individual molecular subtype levels, we discovered distinct characteristics in the K-MASTER cohort, with enrichments of *AKT1-*E17K and *PIK3CA-*H1047R mutations in HR + and HER2 + tumors, respectively. Subsequent comparison of the molecular properties of YBC and OBC patients between K-MASTER and TCGA cohorts elucidated significant enrichments of *NF1* and *ARID1A* mutations in K-MASTER YBC patients, while TCGA OBC predominantly showed mutations in *GATA3* and *CDH1* (Additional file 1: Fig. S4E). Similarly, K-MASTER OBC patients were characterized by a high prevalence of *TP53*, *NF1*, *ARID1A*, and *RB1* mutations, along with genomic amplifications in *ERBB2* and *SRSF2* (Additional file 1: Fig. S4E–F). Notably, mutations in mismatch repair (MMR) encoding molecules, particularly *MSH6*, were more frequently observed in both K-MASTER YBC and OBC patients, which aligned with our previous observations [6]. On the contrary, TCGA OBC patients marked activation of *GATA3* and *CDH1* mutations, as well as copy number alterations in *CCND1*, *FGF3*, *RSF1*, and *GNAS*. Further subgroup analysis revealed that TCGA YBC patients showed frequent genetic alterations in *GATA3*, whereas K-MASTER OBC patients exhibited significant enrichments of *PIK3CA* mutations, highlighting the potential application of *PIK3CA-*mediated therapy. Despite considerable molecular similarities between YBC and OBC in TNBC tumors, K-MASTER OBC patients exhibited a higher frequency of *MSH6* mutations and *SRSF2* amplifications.

### Ethnic-driven molecular disparity in metastatic YBC and OBC patients

**Fig. 2:**
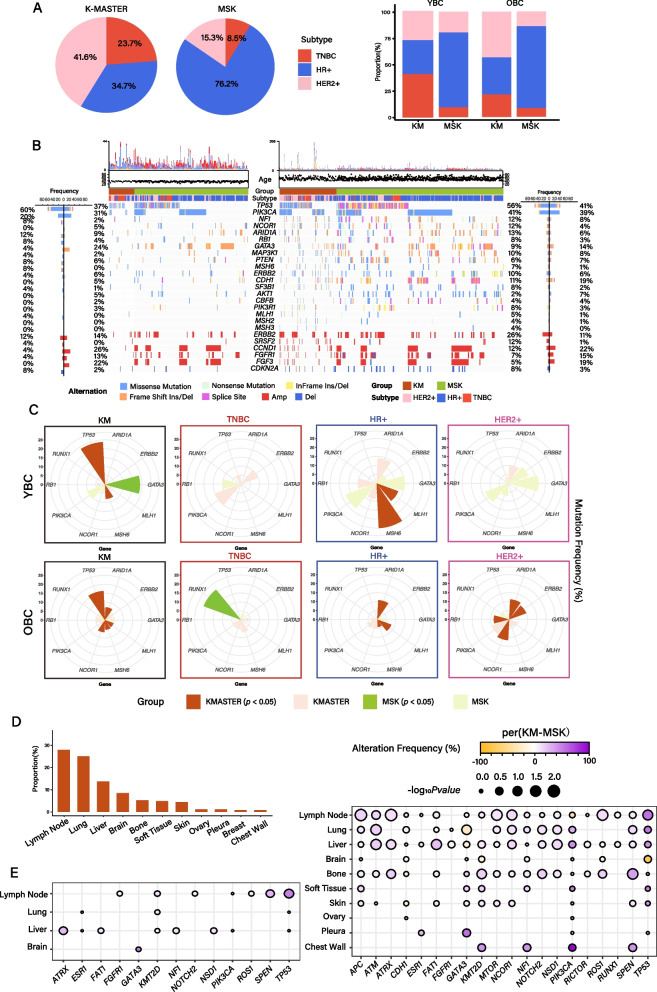
Genomic difference of KM and MSK metastasis cohort. **A** Molecular subtype proportion of KM and MSK (left). Molecular subtype proportion of KM and MSK by age group (right). **B** Genomic landscape of somatic mutations and copy number alterations of YBC BRCA (left) and OBC BRCA (right). **C** Significantly mutated genes according to molecular subtypes in the KM and MSK groups. **D** Metastatic site frequency of KM BRCA metastasis. **E** Mutation frequency difference of KM and MSK by metastatic site

To further investigate the ethnic-driven genomic diversity in YBC and OBC within the metastatic BC context, we curated mutation and copy number alterations data from the MSK-IMPACT cohort. Consistent with primary tumors, metastatic BC from the K-MASTER cohort exhibited a higher proportion of HER2 + and TNBC-type tumors, while MSK-IMPACT was predominantly composed of HR + patients (Fig. 2A). While the MSK-IMPACT cohort demonstrated minimal differences in molecular subtype distribution between YBC and OBC patients, TNBC and HER2 + were the most prominent types in K-MASTER YBC and OBC tumors, respectively (Fig. 2B). The genomic landscape revealed significant molecular differences with K-MASTER YBC characterized by enrichments of *TP53* mutations, while *GATA3* mutations were prominently found in MSK-IMPACT YBC patients (Fig. 2C). Particularly, genes involved in the DNA repair mechanism, including *MLH1*, *MSH6*, and *ARID1A*, were highly mutated in HR + tumors in both K-MASTER YBC and OBC patients (Fig. 2D). While there were no significant ethnic-driven disparities in YBC TNBC and HER2 + tumors, MSK-IMPACT OBC patients exhibited enrichments of *RUNX1* mutations in the TNBC tumors, whereas mutations in *ARID1A*, *ERBB2*, *RB1*, and *NCOR1* were highly prevalent in K-MASTER HER2 + tumors. Examining the chromosomal-level alterations revealed genomic amplifications of *CCND1* and *FGF3* in MSK-IMPACT YBC patients, while in OBC, K-MASTR patients demonstrated genomic amplification in *SRSF2*, *ERBB2*, and *PIK3CA* (Additional file 1: Fig. S5). These results were consistent with our previous comparative analyses on the TCGA patients, highlighting extensive ethnic-driven molecular disparity among YBC and OBC patients.

A substantial number of studies have provided compelling evidence of organotropism, a non-random process where tumor cells preferentially metastasize to specific organs [42]. This complex process is regulated by several key components, including tumor cellular structure, the microenvironment composition of the metastasis destination, and genetic variations [43]. Therefore, to identify ethnic-driven genomic biomarkers associated with organotropism patterns, we meticulously assembled and annotated BC patients, incorporating clinical annotation of all metastatic events and outcomes. Remarkably, lymph node metastasis emerged as the most frequent event, occurring in 28% of all metastatic BC patients, followed by lung (25%), liver (12%), and brain (9%) metastasis (Fig. 2D). Comparative analysis with the MSK-IMPACT cohort revealed substantial ethnic-driven molecular disparities in YBC patients across lymph node, lung, liver, and bone metastases. Specifically, *TP53* mutations exhibited significant enrichment in K-MASTER patients with lymph node, lung, and liver metastases, while MSK-IMPACT patients with lung and brain metastases demonstrated significant associations with *GATA3* and *TP53* mutations, respectively (Fig. 2E). Notably, chest wall metastasis often carried enrichments of *PIK3CA* mutations, which have been previously speculated to promote tumor malignancy and treatment resistance. Interestingly, both ovarian and pleural metastases demonstrated minimal genomic-associated events [44]. Overall, our comprehensive analysis unveiled distinct ethnic- and age-specific associations between genomic aberrations and organotropisms in metastatic breast cancer.

### Ethnic-driven clinical diversity in primary and metastatic YBC and OBC

**Fig. 3:**
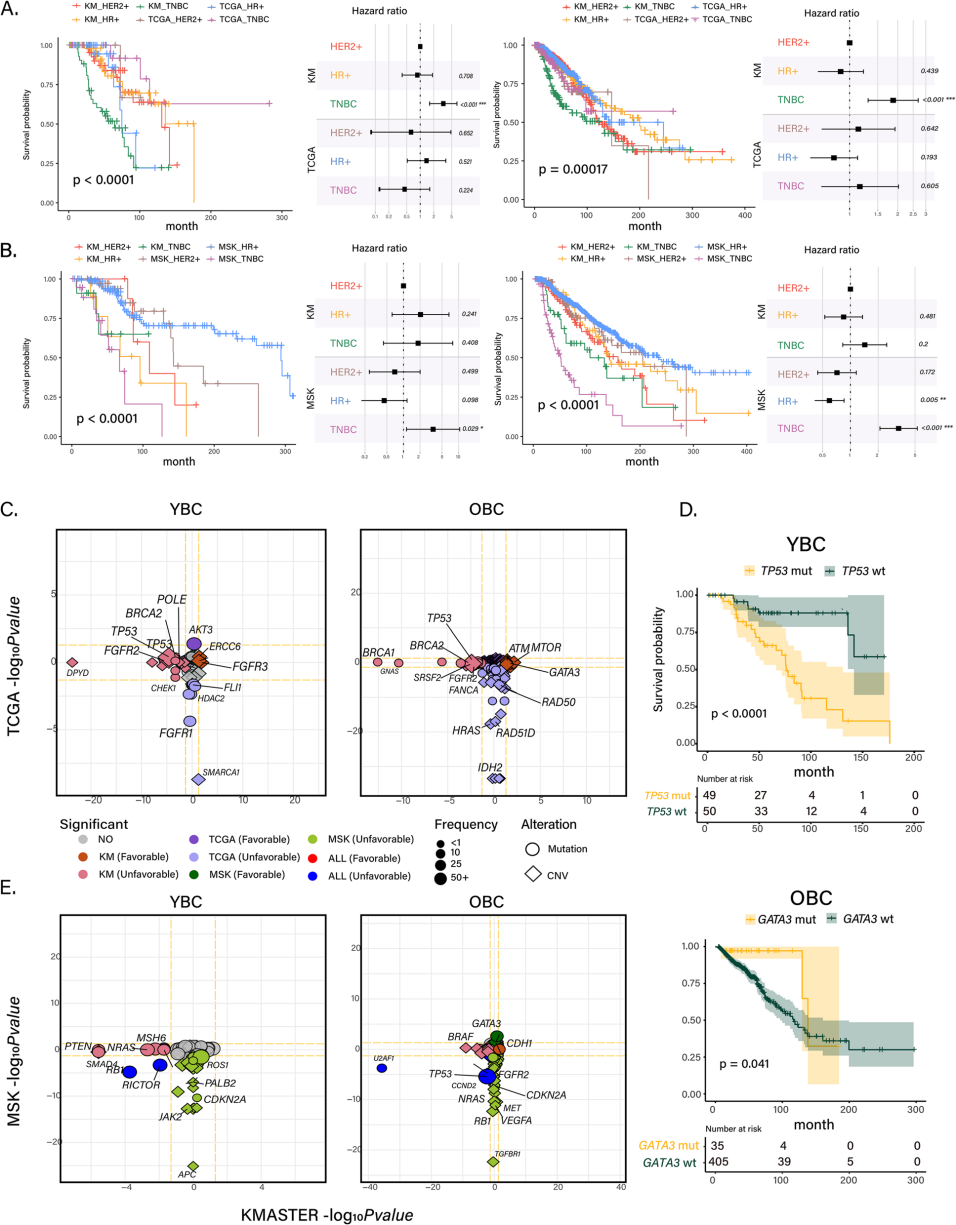
Overall survival analysis of BRCA. **A** Survival analysis of primary YBC K-MASTER and TCGA cohorts (left) and OBC K-MASTER and TCGA cohorts (right). **B** Survival analysis of metastasis K-MASTER and MSK cohorts. Differences in prognosis of primary or metastasis breast cancer in YBC and OBC groups. **C** Finding prognosis marker with somatic mutation and copy number alterations by primary YBC and OBC group in KM and TCGA. **D** Overall survival of primary KM YBC group with/without TP53 mutation (top) and primary KM OBC group with/without GATA3 mutation (bottom). **E** Finding prognosis marker with somatic mutation and copy number alterations by primary YBC and OBC group in KM and TCGA

To interrogate the clinical diversity based on age and ethnicity, we explored the survival probabilities of YBC and OBC patients based on molecular subtypes in K-MASTER, TCGA, and MSK-IMPACT cohorts. Consistent with previous findings, YBC and OBC patients with primary TNBC tumors from the K-MASTER cohort exhibited the worst clinical outcomes (Fig. 3A). In metastatic tumors, TNBC patients from the MSK-IMPACT cohort demonstrated the worst prognosis, while HR + tumors exhibited the most favorable outcomes in both YBC and OBC patients (Fig. 3B). We also observed greater racial differences in the survival outcomes for TNBC tumors in both primary YBC and OBC patients, while metastatic BCs were characterized by significant differences in HR + tumors for YBC and TNBC tumors for OBC patients. We further investigated the molecular characteristics that distinguish survival outcomes in YBC and OBC patients. Interestingly, *AKT3* mutations were significantly associated with improved clinical outcomes, while mutations in *FLI1* and *FGFR1* conferred unfavorable survival probabilities in TCGA YBC patients (Fig. 3C–D). In contrast, *FGFR3*-mutant YBC patients from the K-MASTER cohort showed increased survival probabilities, whereas genetic alterations, including mutation and copy number alterations, in *TP53* were associated with unfavorable clinical outcomes. In OBC, mutations involved in DNA damage repair such as *TP53*, *BRCA1*, *BRCA2*, and *POLE* were enriched in K-MASTER patients with dismal prognoses, while TCGA patients were characterized by chromosomal alterations in *HRAS*, *RAD50*, and *IDH2* (Fig. 3E). Interestingly, several mutations, including *ATM*, *MTOR*, and *GATA3*, demonstrated favorable prognostic effects in K-MASTER OBC patients. For metastatic BC, we discovered that mutations in *RB1* and *RICTOR* were significantly associated with worse clinical outcomes for both K-MASTER and MSK-IMPACT YBC patients, whereas *TP53* and *U2AF1* mutations conferred survival disadvantages in OBC patients from both cohorts. Other prominent genetic associations included mutations in *MSH6*, *NRAS*, and *PTEN* for K-MASTER and *CDKN2A*, *JAK2*, and *APC* genetic alterations for MSK-IMPACT YBC patients with poor clinical outcomes.

### Identification of molecular correlates of therapeutic response to platinum-based chemotherapy

**Fig. 4:**
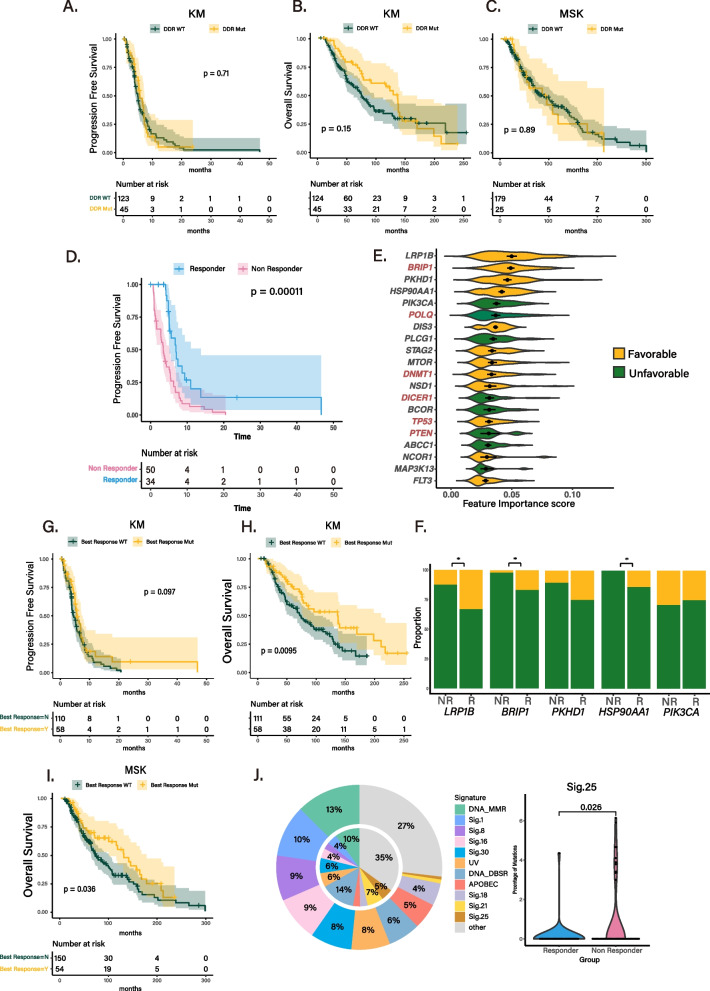
Genomic characteristics analysis of platinum-based chemotherapy treated patient. **A** PFS of platinum-based therapy treated KM with deleterious DDR mutation. **B** OS of platinum-based therapy treated KM with deleterious DDR mutation. **C** OS of platinum-based therapy treated MSK with deleterious DDR mutation. **D** PFS of platinum-based chemotherapy patient by response. **E** Feature important score of genomic alterations by XGBoost. **F** Responder and non-responder group proportion in gene alteration. The asterisks indicate a statistically significant association (*p* < 0.05). **G** PFS of platinum-based therapy treated KM with curated gene set. **H** OS of platinum-based therapy treated KM with curated gene set. **I** OS of platinum-based therapy treated MSK with curated gene set. **J** Mutational signature proportion of responder and non-responder group (left) and signature difference (right)

Platinum-based chemotherapy, including cisplatin and carboplatin, has shown remarkable therapeutic efficacy in patients with metastatic breast cancer [45, 46]. Previous studies have collectively proposed impairments in DNA damage response (DDR) pathways, including homologous recombination deficiency (HRD), and mismatch repair deficiency (MMRd), as diagnostic hallmarks for predicting the treatment response to platinum-based chemotherapy [5, 47, 48]. Therefore, we sought to investigate whether mutations in DDR encoding molecules could potentially serve as surrogate markers for predicting clinical response to platinum therapy. In the KM cohort, 168 BC patients received either cisplatin or carboplatin and among them, 45 patients carried deleterious mutations in DDR genes. Contrary to previous notions, these patients did not demonstrate a favorable clinical response to platinum-based chemotherapy compared to DDR wild-type patients (Fig. 4A–B). We further analyzed the clinical and molecular data from 204 BC patients in the MSK-IMPACT cohort who also received platinum-based chemotherapy, and consistent results were observed (Fig. 4C). Additionally, focusing specifically on HRD-encoding genes yielded similar outcomes (Additional file 1: Fig. S6). Accurate determination of HRD status requires additional information such as loss of heterozygosity, large-scale transition, and telomeric allele imbalances, which are challenging to evaluate within the clinical framework as most practices use targeted sequencing panels such as MSK-IMPACT and FoundationOne [49]. Therefore, to identify a novel molecular signature that could aid in predicting therapeutic response to platinum-based chemotherapy, we employed a multivariable predictive model, XGBoost [50], to assess the significance of individual or combined genomic features. Patients were initially categorized into responders and non-responders based on RECIST criteria, revealing a significant survival difference (Fig. 4D). Subsequently, we constructed a multivariable predictive model using key genomic features that exhibited high mutation rates in both K-MASTER and MSK-IMPACT cohorts. Through hyperparameter optimization and bootstrapping strategy, we obtained robust evaluations of individual mutations as predictive features. As a result, we identified the top 20 molecular features significantly associated with clinical response to platinum-based chemotherapy (Fig. 4E). Notably, mutations in several DDR-related genes, including *BRIP1*, *POLQ*, *DNMT1*, and *DICER1*, demonstrated considerable predictive power. Furthermore, mutations in *LRP1B*, *BRIP1*, *PKHD1*, and *HSP90AA1* were highly enriched in patients showing substantial sensitivity to platinum-based chemotherapy (Fig. 4F). Ultimately, we selected the top 10 genes as the most robust features and established the final parameterized multivariable predictor. Interestingly, K-MASTER patients harboring mutations in the multivariable predictor model exhibited increased clinical response in terms of both progression-free and overall survival (Fig. 4G–H). Moreover, we validated our model using the MSK-IMPACT cohort, where patients carrying genetic alterations in the multivariable model yielded similar results (Fig. 4I). Finally, we compared the mutational signature activities between responders and non-responders and discovered that chemotherapy-associated signature (sig. 25) was highly enriched in non-responder patients (Fig. 4J). Collectively, our findings demonstrated the clinical feasibility of a multivariable predictor model utilizing a prospective sequencing panel to determine the therapeutic response to platinum-based chemotherapy in BC patients.

## Discussion

In this study, we provide a profound insight into the molecular disparities between YBC and OBC patients. Leveraging clinical NGS data from a large collection of 843 East Asian breast cancer patients, we identified significant diversity in key driver genomic alterations and clinical outcomes. Although panel sequencing was conducted without matched normal, we addressed this challenge by excluding mutations predicted to be germline using previously established large databases such as the 1000 Genomes Project, ExAC, and gnomAD. This methodology has been widely employed for clinical analysis [51–53], including our previous study on the comprehensive molecular profiling of 4028 East Asian pan-cancer patients, which yielded profound results [6]. Our findings unveiled notable variations in the prevalence of genetic alterations between YBC and OBC patients. Specifically, YBC patients were more likely to be diagnosed with TNBC tumors with enrichments of *PTEN* and *ARID1A* mutations. Conversely, OBC patients exhibited a high prevalence of HER2 + tumors, accompanied by an increased frequency of activating *PIK3CA* mutations. The extent of molecular disparity became more pronounced when comparing individual molecular subtypes where enrichment of *PTEN* mutations was identified in YBC TNBC tumors, whereas OBC tumors showed chromosomal amplification of *SRSF2*. Additionally, YBC HR + tumors harbored loss-of-function mutations in DNA damage response genes, including *ARID1A* and *PMS2*. In the case of HER2 + tumors, we discovered that OBC patients generally exhibited increased levels of *PIK3CA* mutations, suggesting potential clinical implications of *PIK3CA*-mediated therapy for HER2 + BC patients. Interestingly, we previously presented compelling evidence for clinical response to the PI3K inhibitor, gedatolisib, in a metastatic BC patient with a HER2 + tumor harboring *PIK3CA* mutations, providing a proof-of-concept case [6]. Furthermore, we explored the repertoire of mutational signatures and observed enrichments of signature activities associated with aging and DNA mismatch repair deficiency in both age groups. Specifically, APOBEC signature activity is predominantly observed in YBC patients, particularly within the HR + tumor subtype.

To gain further insights into the distinguishing molecular features of YBC and OBC within different ethnic backgrounds, we conducted a comparative analysis using large-scale mutation and copy number alterations data from both the TCGA and MSK-IMPACT studies. Consistent with previous findings, our results revealed that patients from the K-MASTER cohort were diagnosed at a significantly younger age and exhibited higher proportions of TNBC and HER2 + tumors compared to other cohorts. In particular, K-MASTER YBC patients demonstrated enrichments of *TP53*, *NF1*, *ARID1A*, and *RB1* mutations, while mutations in *PIK3CA* and *GATA3* were predominant in TCGA YBC patients. The presence of *GATA3* mutations in TCGA YBC patients aligned with previous reports indicating improved survival probabilities associated with these mutations [54, 55]. Similarly, in metastatic BC, K-MASTER patients showed a higher incidence of HER2 + and TNBC-type tumors, accompanied by enrichments of *TP53* and *MSH6* mutations, while MSK-IMPACT patients exhibited a higher frequency of *GATA3* mutations. Notably, our investigation of potential associations between genomic alterations and organotropism patterns unveiled considerable ethnic-driven molecular disparities in the lymph node, lung, liver, and bone metastases among YBC patients. Additionally, for OBC patients, we observed that *TP53* mutations conferred increased lymph node metastasis, while *PIK3CA* mutations appeared to be associated with chest wall metastasis in K-MASTER patients, highlighting a potential therapeutic opportunity for PI3K-mediated therapy [56]. These findings provide unprecedented insights into the potential role of genomic alterations in determining organ-specific metastasis patterns in BC and underscore the importance of considering ethnicity in understanding the metastatic process.

Moreover, we conducted an in-depth investigation into the clinical implications of ethnic-driven disparities in YBC and OBC patients. Notably, we observed contrasting clinical outcomes in YBC patients. Consistent with previous studies, the TNBC subtype demonstrated the worst clinical outcomes in K-MASTER YBC patients [57, 58]. However, in TCGA YBC patients, we identified a reverse pattern, where HR + patients demonstrated the worst prognoses. To gain further insights, we examined the molecular characteristics associated with survival outcomes in YBC and OBC patients. Several noteworthy genetic events were identified where *FGFR3* mutations conferred improved clinical outcomes in YBC patients, while mutations in *FLI1* and *FGFR1* were associated with dismal prognoses. Conversely, in OBC patients, mutations in the DNA damage repair genes were generally associated with poor prognoses in the K-MASTER cohort, while chromosomal alterations in *HRAS*, *RAD50*, and *IDH2* were frequently observed in TCGA patients. Lastly, we developed a machine learning-based multivariable model to identify a novel molecular signature capable of predicting treatment response to platinum-based chemotherapy, including cisplatin which is frequently used in the treatment of BC but is difficult to assign due to its high toxicity. Our multivariable predictor model consisted of 10 molecular biomarkers, including *LRP1B*, *BRIP1*, *PKHD1*, and *HSP90AA1*, and demonstrated significant performance in predicting clinical response to platinum-based chemotherapy in K-MASTER patients and was further validated in the MSK-IMPACT cohort.

## Conclusions

Our study provides valuable insights into the understanding of age- and ethnic-driven molecular and clinical disparities in breast cancer patients. By unraveling the intricate relationship between genetic alterations and clinical outcomes, we underscore the potential for personalized treatment strategies in BC patients guided by molecular profiles. Nevertheless, further investigations are warranted to elucidate the underlying mechanisms that govern these dynamic processes. Continued research in this field will pave the way for advancements in tailored therapeutic interventions for various cancer types, including breast cancer.

## Supplementary Information

Additional file 1: Figures S1–S6. Fig. S1 Mutation frequency of KM, Nat Comms 2018 (Samsung Medical Center; SMC) and Cancer Cell 2019 cohort in TNBC, HR+, and HER2+ patients. Fig. S2 Age distribution and somatic alteration in YBC, IBC, and EBC in KM. (A) Age histogram of KM cohort by subtype. (B) Proportion of subtype by age group. (C) Differences in mutation frequency between YBC and EBC group. Fig. S3 (A) Clinical stage distribution of cancer by age and molecular subtype. (B) The OncoKB variant differs from the KM YBC and OBC groups. (C) The OncoKB variant differs from the KM and TCGA groups. (D) The OncoKB variant differs from the KM and MSK groups. Fig. S4 Genomic difference of KM and TCGA primary cohort. (A) Age distribution of KM and TCGA. (B) Molecular subtype proportion of KM and TCGA. (C) Molecular subtype proportion of KM and TCGA by age group. (D) Genomic landscape of somatic mutations and copy number alterations of BRCA by age group. (E) Significantly mutated genes according to molecular subtypes and age group in the KM and TCGA cohorts. (F) Significantly copy number altered genes according to molecular subtypes and age group in the KM and TCGA cohorts. Fig. S5 Gene with significant copy number variation is analyzed according to age and subtype in KM and MSK groups. Fig. S6 PFS of platinum-based therapy treated MSK with deleterious HRD mutation (left) and OS of platinum-based therapy treated MSK with deleterious HRD mutation (right).

Additional file 2: Fig. S1, Table S1. Fig. S1 Mutually exclusivity modules analysis in primary KM and TCGA. (A) Mutation profile of genes belonging to each MEMo module in KM and TCGA. (B) Frequency of alteration in the PI3K/Akt signaling pathway in KM non-TNBC (left box) and KM TNBC (right box). Table S1 Mutually exclusivity modules identified by MEMo in KM.

## Data Availability

The datasets used and/or analyzed during the current study are available from the corresponding author on reasonable request.

## Abbreviations

ER +: Estrogen receptor-positive
HER +: Human epithelial receptor-positive
TNBC: Triple-negative breast cancer
YBC: Young breast cancer
OBC: Older breast cancer
CR: Complete response
PR: Partial response
SD: Stable disease
PD: Progressive disease
PFS: Progression-free survival
CNV: Copy number variations
VAF: Variant allele fraction
MMR: Mismatch repair
DDR: DNA damage response
HRD: Homologous recombination deficiency
