# Supplementary figures and images for: Age- and ethnic-driven molecular and clinical disparity of East Asian breast cancers
Source: BMC Med. 2024 Sep 27;22:422. doi: 10.1186/s12916-024-03638-y (PMC11438198; doi:10.1186/s12916-024-03638-y)

Fig. S1

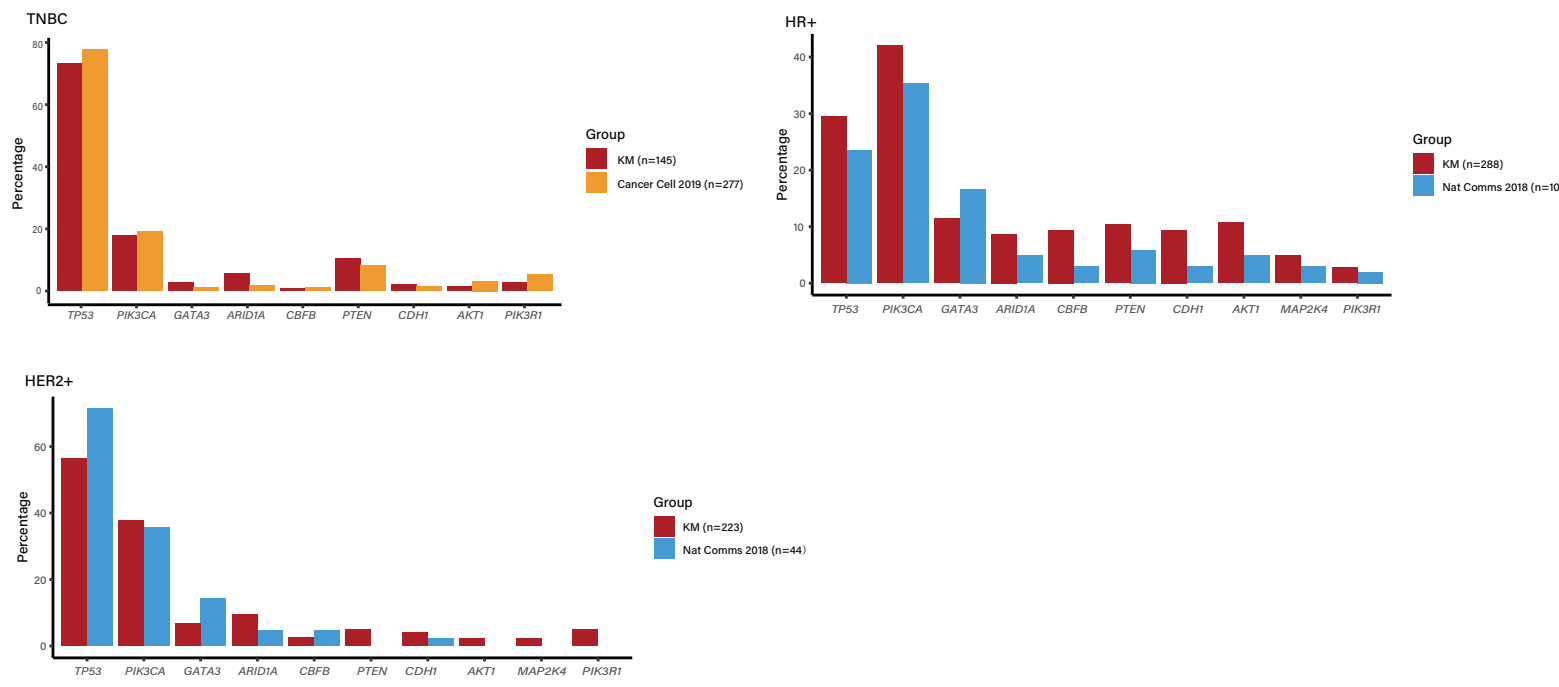

Fig. S2

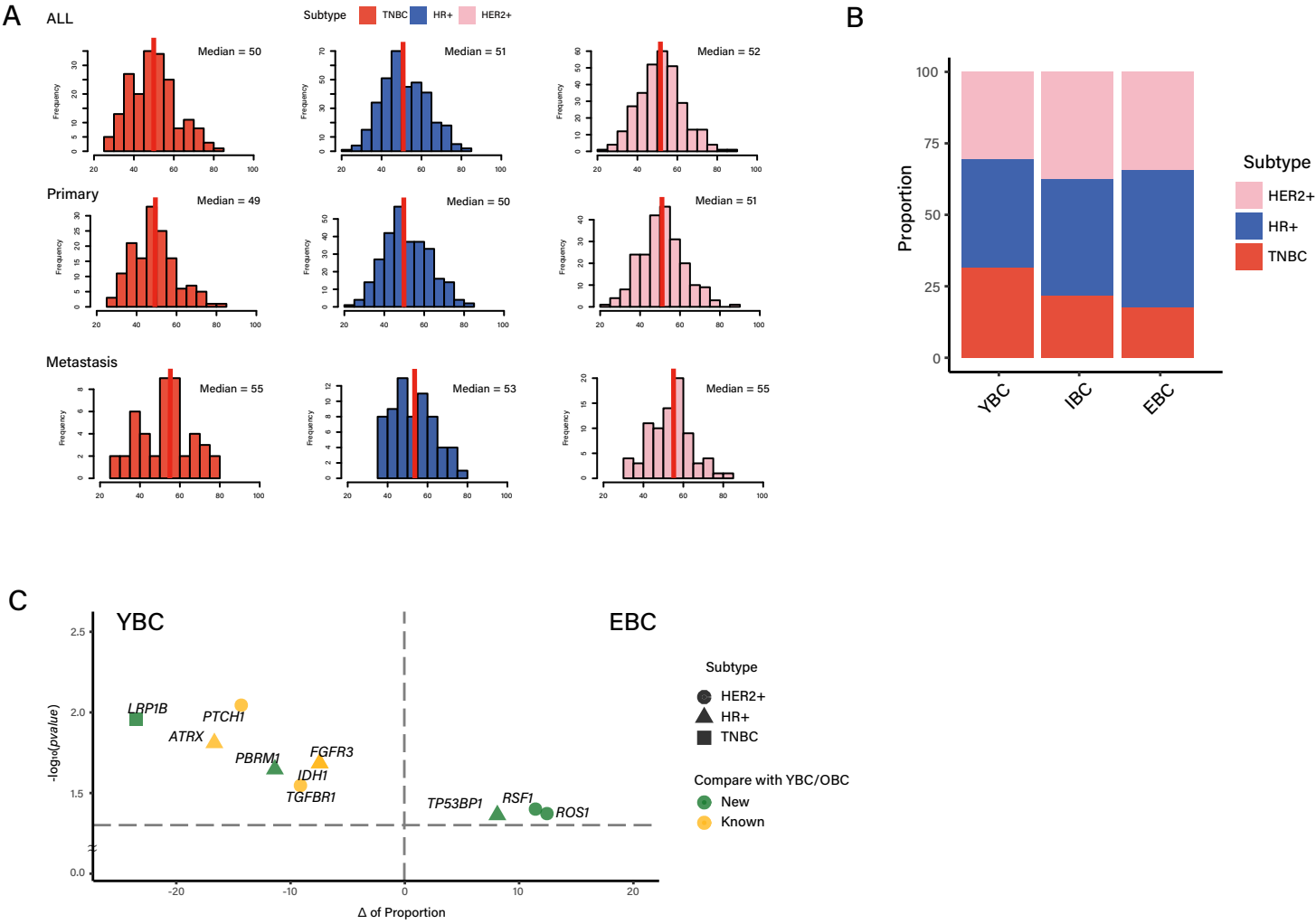

Fig. S3

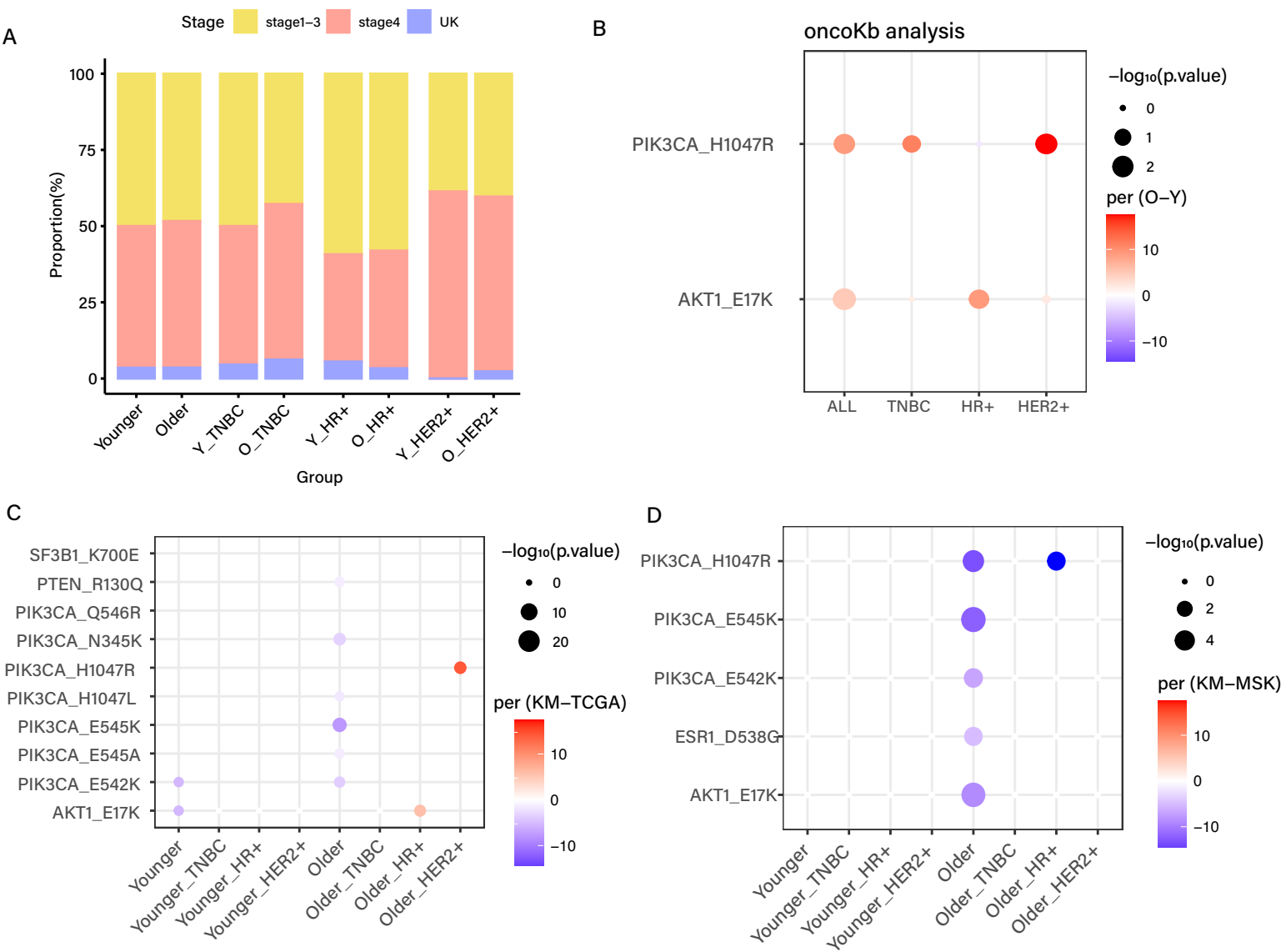

Fig. S4

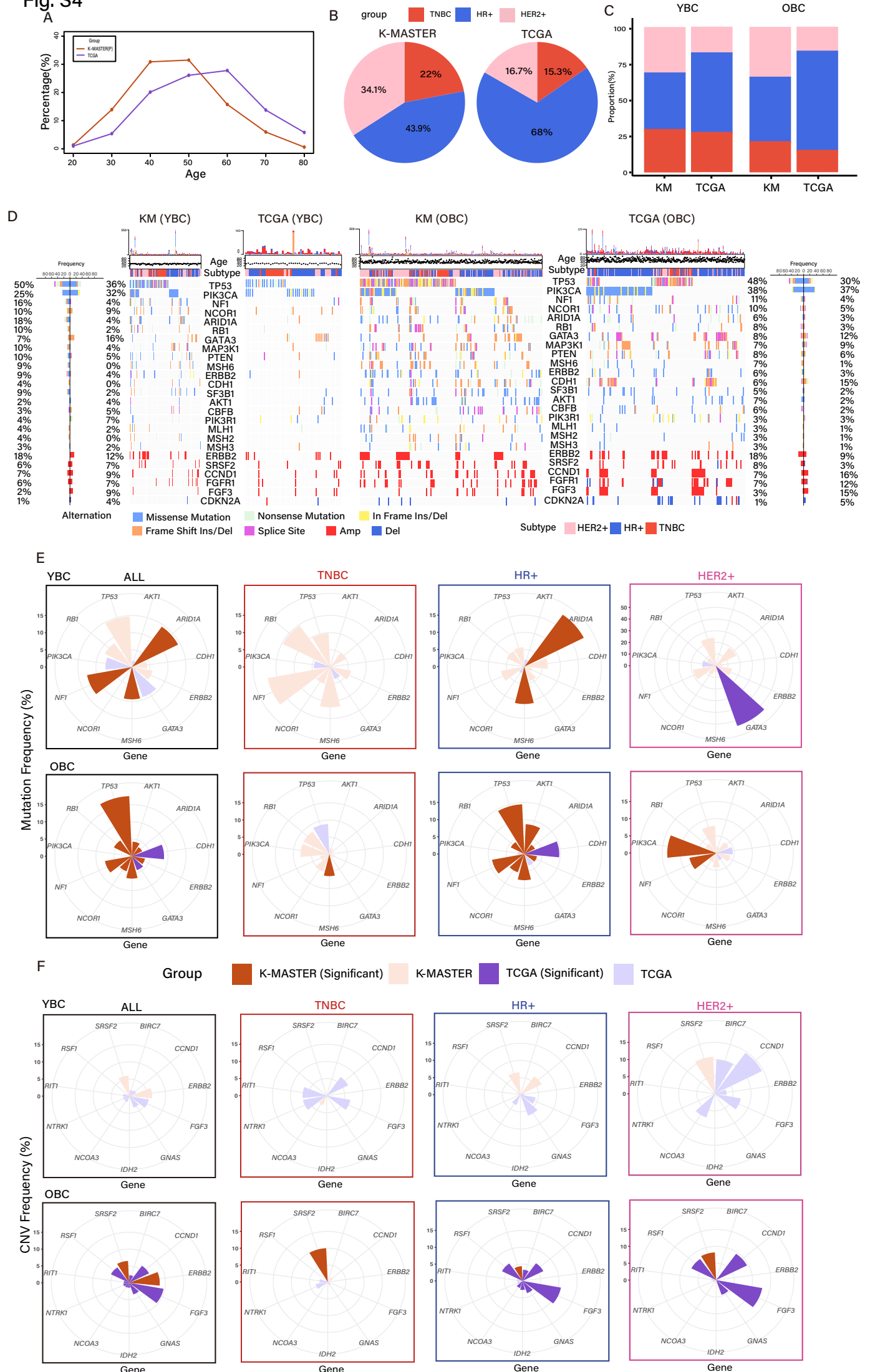

Fig. S5

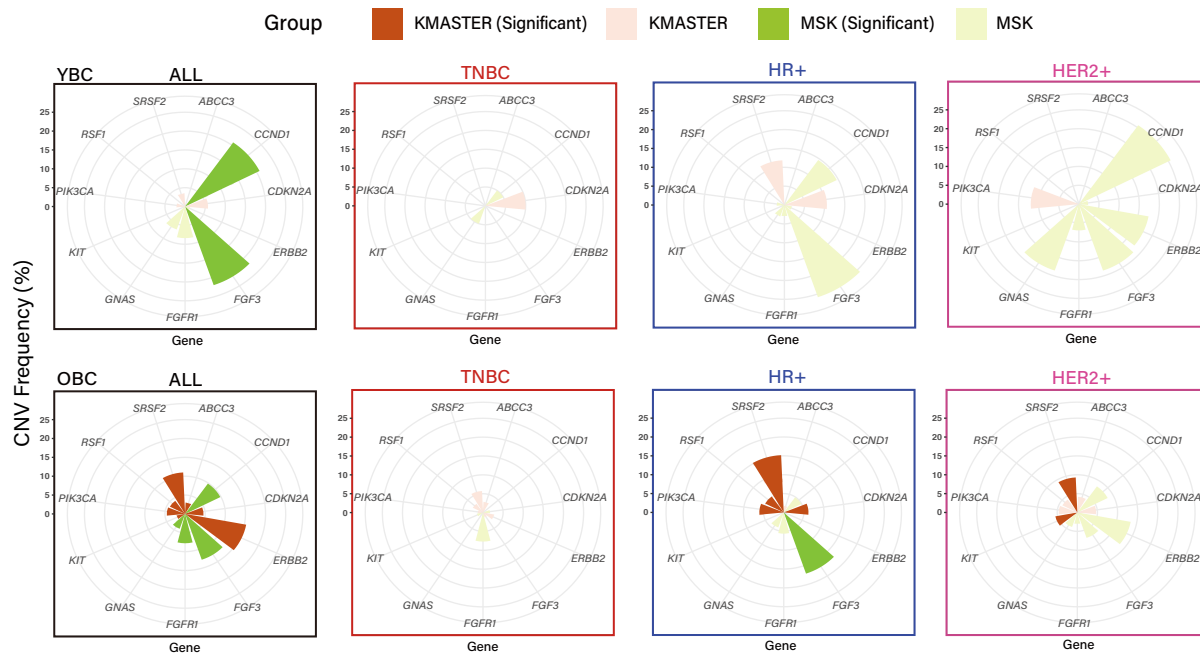

Fig. S6

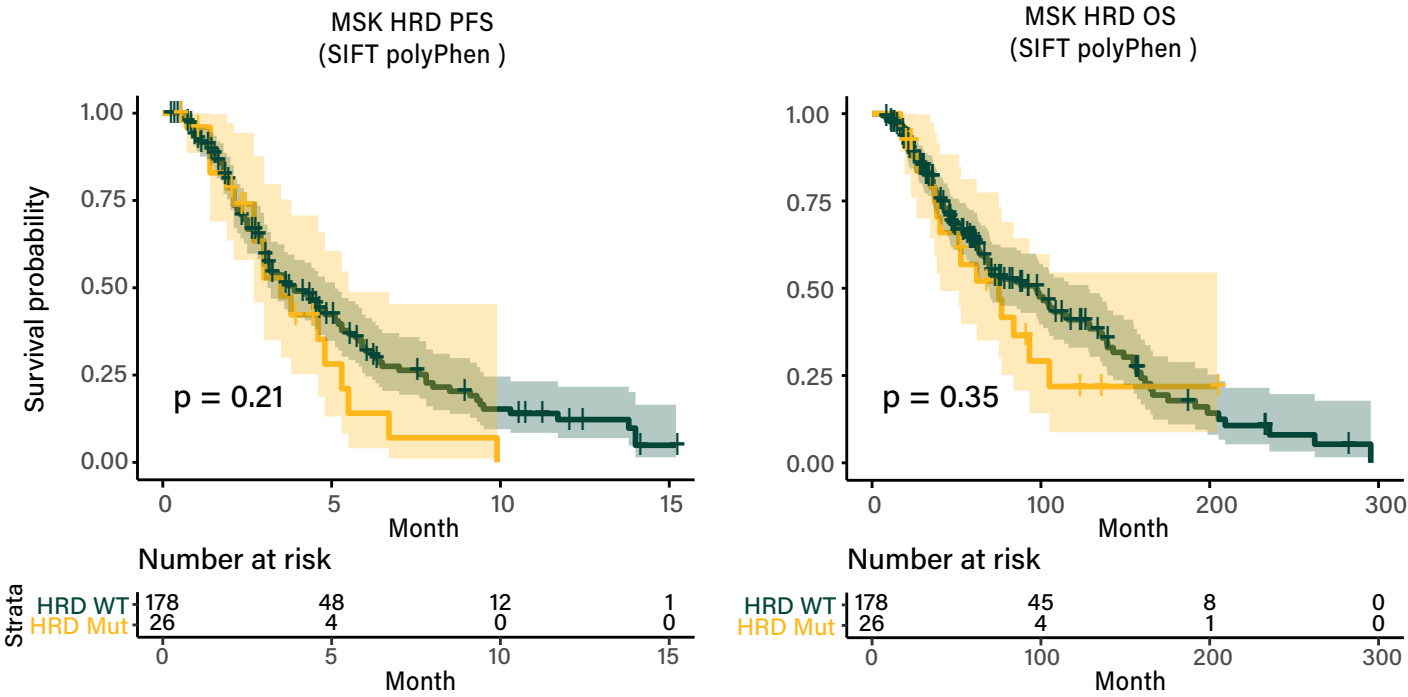

Supplement: Supplementary file 1 — Additional file 1: Figures S1–S6. Fig. S1 Mutation frequency of KM, Nat Comms 2018 (Samsung Medical Center; SMC) and Cancer Cell 2019 cohort in TNBC, HR+, and HER2+ patients. Fig. S2 Age distribution and somatic alteration in YBC, IBC, and EBC in KM. (A) Age histogram of KM cohort by subtype. (B) Proportion of subtype by age group. (C) Differences in mutation frequency between YBC and EBC group. Fig. S3 (A) Clinical stage distribution of cancer by age and molecular subtype. (B) The OncoKB variant differs from the KM YBC and OBC groups. (C) The OncoKB variant differs from the KM and TCGA groups. (D) The OncoKB variant differs from the KM and MSK groups. Fig. S4 Genomic difference of KM and TCGA primary cohort. (A) Age distribution of KM and TCGA. (B) Molecular subtype proportion of KM and TCGA. (C) Molecular subtype proportion of KM and TCGA by age group. (D) Genomic landscape of somatic mutations and copy number alterations of BRCA by age group. (E) Significantly mutated genes according to molecular subtypes and age group in the KM and TCGA cohorts. (F) Significantly copy number altered genes according to molecular subtypes and age group in the KM and TCGA cohorts. Fig. S5 Gene with significant copy number variation is analyzed according to age and subtype in KM and MSK groups. Fig. S6 PFS of platinum-based therapy treated MSK with deleterious HRD mutation (left) and OS of platinum-based therapy treated MSK with deleterious HRD mutation (right). [file 12916_2024_3638_MOESM1_ESM.pdf]
