# Supplementary material for: Age- and ethnic-driven molecular and clinical disparity of East Asian breast cancers
Source: BMC Med. 2024 Sep 27;22:422. doi: 10.1186/s12916-024-03638-y (PMC11438198; doi:10.1186/s12916-024-03638-y)
Supplement: Supplementary file 2 — Additional file 2: Fig. S1, Table S1. Fig. S1 Mutually exclusivity modules analysis in primary KM and TCGA. (A) Mutation profile of genes belonging to each MEMo module in KM and TCGA. (B) Frequency of alteration in the PI3K/Akt signaling pathway in KM non-TNBC (left box) and KM TNBC (right box). Table S1 Mutually exclusivity modules identified by MEMo in KM. [file 12916_2024_3638_MOESM2_ESM.pdf]

Fig. S1

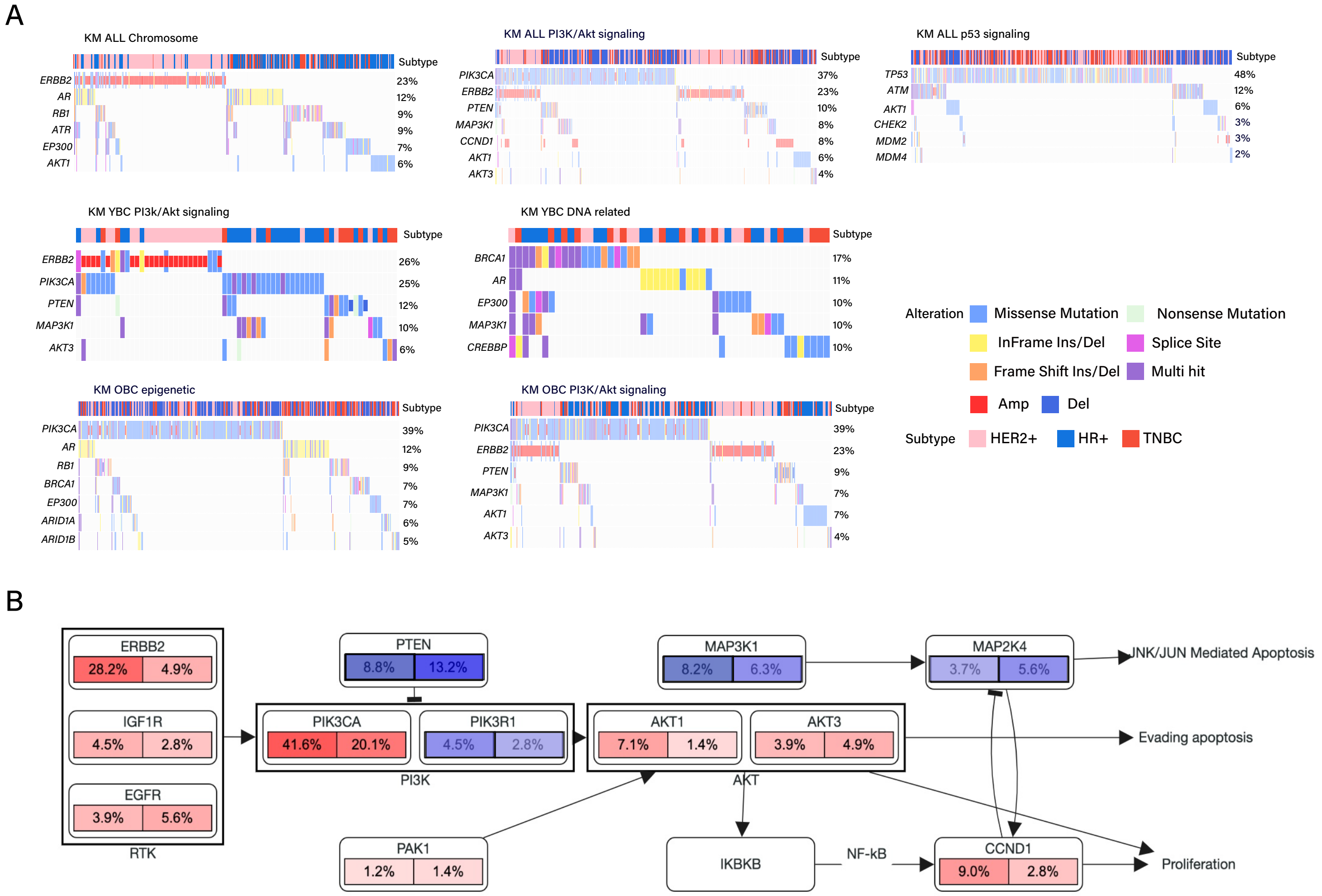

Table S1

Module ID	Genes	Total Percent Altered Cases	p-value	p*-value
M1	AKT1 [38], AR [78], BRCA1 [57], CREBBP [51], EP300 [48], RB1 [58],	38.38%	0	0.06
M2	AKT1 [38], AR [78], BRCA1 [57], CREBBP [51], MAP3K1 [51], RB1 [58],	37.77%	0	0.06
M3	AKT1 [38], AR [78], ATR [57], BRCA1 [57], EP300 [48], RB1 [58],	37.31%	0	0.06
M4	AKT1 [38], AR [78], BRCA1 [57], EP300 [48], NCOR1 [65], RB1 [58],	38.84%	0	0.06
M5	AKT1 [38], AR [78], EP300 [48], ERBB2 [151], RB1 [58],	45.57%	0	0.06
M6	AKT1 [38], AR [78], ERBB2 [151], MAP3K1 [51], RB1 [58],	46.18%	0	0.06
M7	AR [78], ERBB2 [151], NF1 [80], PTEN [63], RB1 [58],	49.69%	0	0.06
M8	AKT1 [38], AR [78], ERBB2 [151], PTEN [63], RB1 [58],	48.62%	0	0.06
M9	AKT1 [38], AR [78], BRCA1 [57], EP300 [48], ERBB2 [151],	45.26%	0	0.06
M10	AR [78], ARID1A [54], BRCA1 [57], CREBBP [51], EP300 [48],	33.64%	0	0.06
M11	AKT1 [38], AR [78], BRCA1 [57], ERBB2 [151], MAP3K1 [51],	44.95%	0	0.06
M12	AKT1 [38], AR [78], BRCA1 [57], ERBB2 [151], RB1 [58],	46.48%	0	0.06
M13	AR [78], ARID1A [54], BRCA1 [57], NF1 [80], RB1 [58],	37.00%	0	0.06
M14	AKT1 [38], AR [78], BRCA1 [57], PIK3CA [231], RB1 [58],	57.19%	0	0.06
M15	AKT1 [38], BRCA1 [57], ERBB2 [151], MAP3K1 [51], RB1 [58],	43.12%	0	0.06
M16	AR [78], ARID1A [54], EP300 [48], NCOR1 [65], RB1 [58],	35.17%	0	0.06
M17	AKT1 [38], AR [78], ERBB4 [41], KIT [40], PTEN [63],	32.42%	0	0.06
M18	AKT1 [38], AR [78], ERBB2 [151], PIK3CA [231],	60.24%	0	0.06
M19	AKT1 [38], CREBBP [51], EP300 [48], ERBB2 [151],	36.39%	0	0.06
M20	AKT1 [38], AR [78], EP300 [48], PIK3CA [231],	51.99%	0	0.06
M21	AKT1 [38], AR [78], ERBB2 [151], KIT [40],	39.76%	0	0.06
M22	AKT1 [38], ERBB2 [151], PIK3CA [231], RB1 [58],	58.41%	0	0.06
M23	AKT1 [38], PIK3CA [231], PTEN [63], RB1 [58],	49.54%	0	0.06
M24	AKT1 [38], BRCA1 [57], ERBB2 [151], PIK3CA [231],	58.26%	0	0.06
M25	AKT1 [38], BRCA1 [57], CREBBP [51], PIK3CA [231],	49.24%	0	0.06
M26	AKT1 [38], AR [78], BRCA1 [57], KIT [40],	27.52%	0	0.06
M27	AKT1 [38], AR [78], ERBB2 [151], ERBB4 [41],	39.14%	0	0.06
M28	AR [78], NF1 [80], PIK3CA [231],	50.15%	0	0.06
M29	BRCA1 [57], NF1 [80], PIK3CA [231],	47.40%	0	0.06
M30	AKT1 [38], ERBB4 [41], PIK3CA [231],	43.12%	0	0.06
M31	AR [78], ARID1A [54], BRCA1 [57], EP300 [48], RB1 [58],	34.56%	0.001	0.06
M32	AKT1 [38], AR [78], CREBBP [51], EP300 [48], NCOR1 [65],	33.18%	0.001	0.06
M33	AR [78], ARID1A [54], NCOR1 [65], NF1 [80], RB1 [58],	37.61%	0.001	0.06
M34	ARID1A [54], BRCA1 [57], EP300 [48], GATA3 [52], NCOR1 [65],	31.96%	0.001	0.06
